# Housing Systems Influence Gut Microbiota Composition of Sows but Not of Their Piglets

**DOI:** 10.1371/journal.pone.0170051

**Published:** 2017-01-13

**Authors:** Tereza Kubasova, Lenka Davidova-Gerzova, Elodie Merlot, Matej Medvecky, Ondrej Polansky, Delphine Gardan-Salmon, Helene Quesnel, Ivan Rychlik

**Affiliations:** 1 Veterinary Research Institute, Hudcova, Brno, Czech Republic; 2 INRA, UMR1348, PEGASE, Saint-Gilles, France; 3 CCPA Group, Z.A. du Bois de Teillay, Janze, France; Wageningen University, NETHERLANDS

## Abstract

Different housing systems can be used in pig production and little is known about their effect on gut microbiota composition. In this study we characterized fecal microbiota by sequencing the rRNA genes in sows kept during gestation in conventional pens with a slatted floor and in enriched pens with a floor covered with deep straw. After farrowing, microbiota of 1- and 4-day-old piglets were also monitored. Microbiota of sows from the enriched system contained significantly more *Prevotella*, *Parabacteroides*, CF231, *Phascolarctobacterium*, *Fibrobacter*, *Anaerovibrio* and YRC22 and significantly less *Lactobacillus*, *Bulleidia*, *Lachnospira*, *Dorea*, *Ruminococcus* and *Oscillospira* than microbiota of sows from the conventional system. The *Firmicutes* to *Bacteroidetes* ratio was 0.96 in the microbiota of sows kept in the enriched pens and this increased to 1.66 in the microbiota of sows kept in the conventional system. The production system therefore influenced microbiota composition, most likely due the ingestion of the straw. The microbiota of 1- and 4-day-old piglets differed from the microbiota of sows and sows therefore did not represent the most important source for their colonization in early days of life.

## Introduction

Gut microbiota play an important role for its host. Butyrate produced by commensal microbiota is the most preferred energy source for colonocytes [1]. Microbiota also degrade complex polysaccharides and bacterial species belonging to the genus *Bacteroides* forage on host proteins forming the mucus above epithelial cells [2]. Beneficial species in gut microbiota prevent the multiplication of pathogens by simple competition for available nutrients. Finally, gut microbiota stimulates the immune system thus playing a crucial role in neonate immune system maturation [3]. Correct colonization of the intestinal tract, in the early days of life in particular, is therefore of utmost importance for any individual and the quality of this early colonization may determine the extent of digestive disorders occurring during the first weeks of life or affect long term intestinal homeostasis [4,5].

Colonization of the intestinal tract in piglets is initiated at birth by microbiota from the maternal genital and intestinal tract. Soon after, colonization of the intestinal tract of newborns is complemented by microbiota from the environment. Depending on management and production conditions, the importance of maternal and environmental microbiota sources may vary considerably. In pig production, the cleaning procedures applied in the farrowing unit prior to entrance of the sows decrease the occurrence of environmental microbiota. Despite this, it is difficult to predict to what extent the sows are responsible for the development of microbiota in piglets. There is a report showing that administration of probiotics to sows during gestation affects piglet microbiota composition suggesting a maternal influence [6]. On the other hand, a higher similarity of microbiota between piglets co-inhabiting the same pen in comparison to separated siblings rather favors the environmental effect [7].

Various production systems, which may affect gut microbiota composition, are used in pig production. One example is represented by the conventional and organic farming systems. However, when microbiota composition in pigs in conventional or organic farming was compared, there were no extensive differences and microbiota of conventional and organic pigs were alike [8]. Similarly, no significant differences in the composition of microbiota of indoor and outdoor kept pigs were reported [9]. Various systems are in use also for rearing sows during gestation. The most widespread production system is characterized by a simple slatted floor but alternative enriched systems with deep straw bedding are also in use. In the latter system, lower stress and the consumption of straw containing a non-digestible fiber may have an impact on gut microbiota [10, 11]. To address this hypothesis, here we characterized the fecal microbiota of sows kept in conventional and enriched pens during gestation. In addition, we investigated whether a maternal housing system may affect the development of microbiota in piglets during their first week of life.

## Material and Methods

### Ethical statement

The experiment was carried out at the experimental farm of Creécom (Chambre d’Agriculture de Bretagne, France). Animals were reared following French guidelines for animal care and use. The experimental protocol was approved by the Local Ethics Committee for Animal Experiments in Rennes (Comité Rennais d'Ethique en matière d'Expérimentation Animale), France, and the French Ministry of Higher Education and Research (agreement n° 02805.02). All sampled animals were returned back to the commercial production at the end of the experiment.

### Animals and experimental design

Large-White x Landrace sows were divided into 2 independent farrow-to-finish units with identical management strategies, but differing in the housing environment for gestating sows. Pens in the conventional unit were furnished with a concrete slatted floor with 2.4 m^2^ space per sow, and pens in the enriched unit were covered with deep straw with more space per animal (3.4 m^2^ per sow). All sows were transferred to identical individual farrowing crates (2.25 x 0.5 m) with a slatted floor at gestation day 105. When needed, piglet adoptions were performed among litters of the same treatment group to equalize the size of the litters. Common procedures (iron injection, tooth grinding, tail resection, castration) were applied between 24 and 72 hours after birth. Sows were fed a standard gestation and lactation diet.

The experiment was repeated on 3 replicates with 3 different batches of sows that farrowed in April 2014, September 2014 and January 2015. Fecal samples were collected from sows during farrowing (sows originating from the conventional system: n = 6, 6 and 6, and from the enriched system: n = 6, 8 and 7, in batch 1, 2 and 3, respectively). Fecal samples were collected from piglets on day 1 of life in the first replicate (13 piglets of the 6 sows from the conventional system and 11 piglets of the 6 sows from the enriched system). Fecal samples were also collected from 4-day-old piglets (n = 9, 13 and 16 piglets of sows the conventional system and 9, 15 and 14 piglets of sows from the enriched pens in batch 1, 2 and 3, respectively). Sample collection on day 1 was performed before starting the adoption procedures, and piglets sampled on day 4 were pigs that remained with their mother from birth (i.e. non-adopted piglets) although some piglets in their litter could have originated from another biological mother and thus may have brought a different flora. Altogether 139 fecal samples (39 samples from sows, 24 from 1-day-old piglets and 76 from 4-day-old piglets) were collected and analyzed. Immediately after collection into a cryotube, fecal samples were frozen in liquid nitrogen and kept at -80°C. Age of sampled piglets was selected as the time point when caused of piglet mortality shift from nutritional (starvation) and accidental (crushing) causes to infectious causes, including digestive disorders and diarrhea.

### Microbiota characterization

Fecal samples were homogenized using zirconia silica beads (BioSpec Products) in a MagNALyzer (Roche Diagnostics). Following homogenization, the DNA was extracted using the QIAamp DNA Stool Mini Kit according to the manufacturer’s instructions (Qiagen). The DNA concentration was determined spectrophotometrically and the DNA was stored at −20°C until use. Prior to PCR, DNA samples were diluted to 5 ng/μl and used as a template in PCR with forward primer 5′-*TCGTCGGCAGCGTCAGATGTGTATAAGAGACAG*-MID-GT-CCTACGGGNGGCWGCAG-3′ and reverse primer 5′-*GTCTCGTGGGCTCGGAGATGTGTATAAGAGACAG*-MID-GT-GACTACHVGGGTATCTAATCC-3′. The sequences in italics served as an index and adapter ligation while underlined sequences allowed for amplification over V3/V4 region of eubacterial 16S rRNA genes. MIDs represent different sequences of 5, 6, 9 or 12 bp in length designed to differentiate samples. PCR amplification and clean up were performed using the KAPA Taq HotStart PCR kit (Kapa Biosystems). In the next step the concentration of PCR products was determined spectrophotometrically, the DNA was diluted to 100 ng/μl and groups of 14 PCR products with different MID sequences were indexed with a Nextera XT Index Kit following the manufacturer’s instructions (Illumina). Prior to sequencing, the concentration of differently indexed samples was determined using a KAPA Library Quantification Complete kit (Kapa Biosystems). All indexed samples were diluted to 4 ng/μl and of phiX DNA was added to 20% final concentration. Sequencing was performed using MiSeq Reagent Kit v3 and MiSEQ 2000 apparatus according to the manufacturer’s instructions (Illumina).

The fastq files generated after Illumina sequencing were uploaded into Qiime software [12]. Quality trimming criteria were set to a value of 19 and no mismatch in the MID sequences. Reverse reads were shortened to a length of 250 bp and forward and reverse sequences were joined. Chimeric sequences were predicted by the slayer algorithm and excluded from subsequent analysis. The resulting sequences were then classified by RDP Seqmatch with an OTU (operational taxonomic units) discrimination level set to 97% followed by UniFrac analysis. Principal coordinate analysis (PCoA) implemented in Qiime was used for data visualization. The raw sequence reads have been deposited in the NCBI Short Read Archive under the accession number SRP071000.

### Comparison of microbiota abundance in animals kept in conventional and enriched systems

Only bacterial genera which were present in more than 90% of tested samples in at least one of the tested categories, *i*.*e*. sows, 1-day-old and 4-day-old piglets, were included in this analysis. This selection was adopted to avoid false positive conclusions due to quite unequal coverage of individual samples. Focusing on common microbiota members permitted the use of a t-test applied on the percentual representation of each genus in the population. Comparisons with p<0.05 were considered as significant. Hierarchical clustering was calculated at genus level using genera for which at least 5 reads were available after summing up reads from all samples. This resulted in 189 genera for which percentual representation in each sample was calculated. This table was used for the calculation of distance matrix was using Euclidean distance measure. Hierarchical clustering was then prepared using Ward's linkage method.

## Results

### Fecal microbiota of sows, 1- and 4-day-old piglets

**Fig 1:**
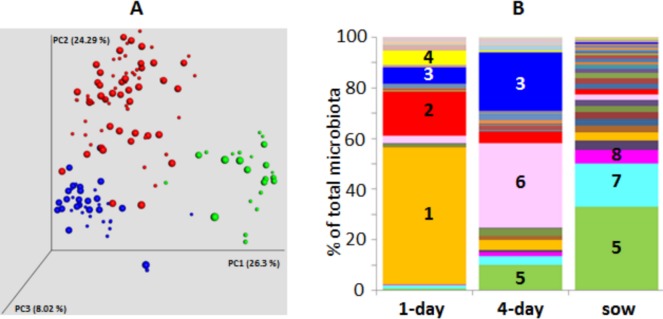
Microbiota composition in sows, 1- and 4-day-old piglets. Panel A, weighted PCoA analysis of microbiota composition in sows (blue spots), 1-day-old piglets (green spots) and 4-day-old piglets (red spots). Smaller spots–microbiota of sows kept under conventional conditions with slatted floor, or of piglets delivered by these sows. Bigger spots—microbiota of sows kept in enriched pens with floor covered with straw bedding, or of piglets delivered by these sows. Panel B, composition of fecal microbiota of 1-day-old piglets, 4-day-old piglets and sows at the time of farrow 1—*Escherichia*, 2 –*Clostridium*, 3—*Fusobacterium*, 4—*Actinobacillus*, 5—*Prevotella*, 6 –*Bacteroides*, 7—*Oscillospira*, 8—*Ruminococcus*. For all genera, see S1 Table.

**Fig 2:**
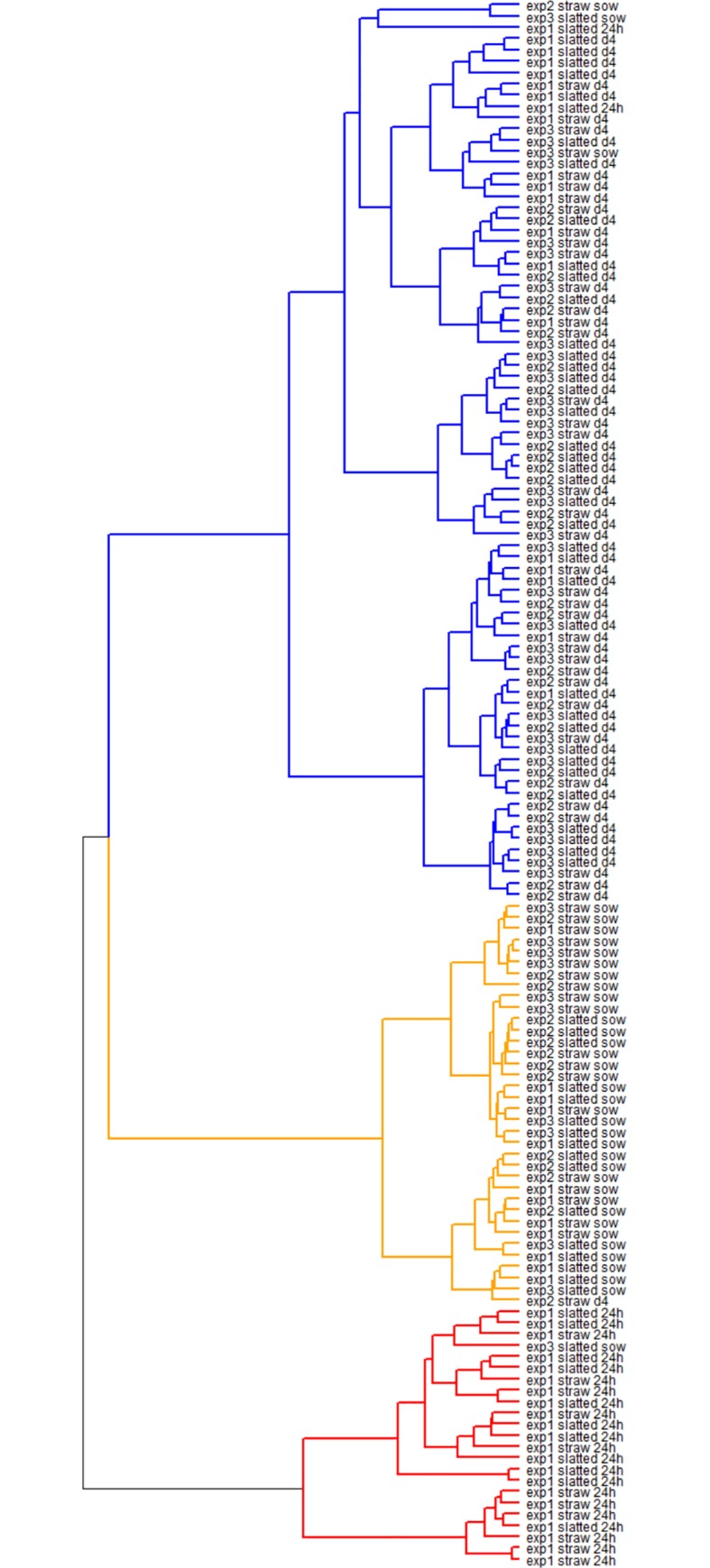
Hierarchical cluster analysis of microbiota composition in sows, 1- and 4-day-old piglets. Samples are identified by experiment followed by rearing conditions (slatted -conventional conditions with slatted floor, straw—enriched pens with floor covered with straw bedding), and age category (sow, 1-day-old and 4-day-old piglet).

Altogether 3,404,413 reads were obtained for all the samples. Median read count per sample was 13,411 ranging from 406 reads in the sample with the lowest coverage to 173,766 reads in the sample with the highest coverage. Species abundance and diversity increased in a direction 1-day-old piglets—4-day-old piglets—adult sows. However, comparison of species abundances or diversity indices in animals from conventional or enriched conditions never resulted in statistical significance (Table 1). PCoA clustering showed that microbiota of sows, 1- and 4-day-old piglets differed considerably (Fig 1A). Although there was pig-to-pig variation in all age categories, the lowest variation was recorded in the composition of microbiota of sows which formed a well-defined cluster while microbiota of 1- and 4-day old piglets were subjected to greater variation since the two clusters were of a more diffuse shape. A hierarchical cluster analysis based on the detected genera confirmed PCoA clustering and also excluded any “experiment” effect since sows and 4-day-old piglets from all 3 experiments were randomly mixed up (Fig 2). Two main clusters were formed within sow microbiota. The first one comprised 22 samples and 8 of these originated from the sows kept under conventional conditions. The second major cluster comprised 13 samples and 8 of these originated from the sows kept under conventional conditions. The second cluster was therefore weakly enriched for sows kept under conventional conditions.

**Table 1:** Basic diversity characteristics of microbiota present in feces of each category of pigs analyzed in this study.

	1-day-old piglets		4-day-old piglets		sows	
	conventional	enriched	conventional	enriched	conventional	enriched
observed species	478±360	720±463	814±438	739±406	2788±1517	2746±3636
chao1 species estimate	1163±678	1978±1367	2316±1316	1977±1149	8640±5688	8769±12528
Shannon’s index	3.79±1.07	2.91±1.22	5.28±0.66	5.10±0.70	7.70±0.96	8.02±1.04
Simpson‘s index	0.77±0.15	0.62±0.23	0.91±0.04	0.90±0.05	0.97±0.03	0.97±0.06

Altogether 36 different genera were found in microbiota of at least 90% of sows, *i*.*e*. in at least 35 out of 39 tested sows. *Prevotella* (phylum *Bacteroidetes*) and *Oscillospira* (phylum *Firmicutes*) dominated over the remaining genera and formed nearly 50% of the total microbial population in the faeces of sows at the time of farrowing (Fig 1B). Microbiota of 1-day-old piglets was dominated by *Escherichia* which formed over 50% of total microbiota. An additional 3 genera characteristic of 1-day-old piglets included *Clostridium*, *Fusobacterium* and *Actinobacillus* which together with *Escherichia* formed over 80% of all microbiota. The most abundant microbiota members of 4-day-old piglets included *Bacteroides*, *Fusobacterium* and *Prevotella*. These 3 genera formed over 60% of total microbiota. *Bacteroides* formed 30% and *Prevotella* 10% of microbiota in 4-day-old piglets whilst in sows, *Prevotella* increased to 24% and *Bacteroides* decreased to only 1.8% of total microbiota (Fig 1B).

### Comparison of microbiota abundance in animals kept under conventional and enriched systems

PCoA and hierarchical cluster analysis indicated a minor separation of sows kept under conventional and enriched conditions (Figs 1A and 2). In the next step we therefore directly compared the abundance of individual genera in sows kept under different conditions. This analysis showed that 13 genera were differently abundant, 6 being more abundant in the microbiota of sows kept under conventional conditions and 7 being more abundant in microbiota of sows kept under enriched conditions with straw bedding. Although the differences in abundance were only between 1.34 to 5 fold, significant differences were recorded among common microbiota members including the two the most abundant genera, *i*.*e*. *Prevotella* and *Oscillospira* (Table 2). We also noticed that all genera being of a higher abundance in sows from conventional conditions belonged to the phylum *Firmicutes* whilst genera of higher abundance in microbiota in sows kept under enriched conditions belonged mainly to the phyla *Bacteroidetes* or *Fibrobacteres*. The *Firmicutes* to *Bacteroidetes* ratio in microbiota from sows from conventional conditions was 1.66 whilst this ratio decreased to 0.96 in microbiota of sows kept under enriched conditions with straw bedding.

**Table 2:** Bacterial genera differently represented (p<0.05) in sows kept under conventional and enriched conditions, and in their piglets.

Genus	Phylum	Category	Conventional (% of total microbiota)	Enriched (% of total microbiota)	Conv/Enrich ratio
*Lactobacillus*	*Firmicutes*	sow	5.33	1.08	4.94
*Bulleidia*	*Firmicutes*	sow	0.55	0.25	2.25
*Lachnospira*	*Firmicutes*	sow	0.29	0.14	2.03
*Dorea*	*Firmicutes*	sow	1.47	0.86	1.70
*Ruminococcus*	*Firmicutes*	sow	6.35	3.94	1.61
*Oscillospira*	*Firmicutes*	sow	19.60	13.01	1.51
*Prevotella*	*Bacteroidetes*	sow	27.73	37.25	0.74
*Parabacteroides*	*Bacteroidetes*	sow	1.46	2.55	0.57
*CF231*	*Bacteroidetes*	sow	2.01	3.61	0.56
*Phascolarctobacterium*	*Firmicutes*	sow	1.51	3.01	0.50
*Fibrobacter*	*Fibrobacteres*	sow	0.08	0.17	0.44
*Anaerovibrio*	*Firmicutes*	sow	0.83	2.08	0.40
*YRC22*	*Bacteroidetes*	sow	0.22	0.75	0.29
*Streptococcus*	*Firmicutes*	1d piglet	2.02	0.43	4.71
*Escherichia*	*Proteobacteria*	1d piglet	35.60	58.55	0.61
*Veillonella*	*Firmicutes*	4d piglet	1.56	0.55	2.84
*Pasteurella*	*Proteobacteria*	4d piglet	1.60	0.78	2.06

A lower number of differently represented genera was recorded in 1- and 4-day-old piglets. *Streptococcus* was more frequent in microbiota of 1-day-old piglets from sows housed in conventional conditions and *Escherichia* was more abundant in microbiota of 1-day-old piglets from sows housed in the enriched production system. *Veillonella* and *Pasteurella* were more frequent in microbiota of 4-day-old piglets from sows housed in the conventional production system (Table 2).

## Discussion

In this study, we analyzed the fecal microbiota composition in sows kept under 2 different production systems during gestation and in their piglets. *Escherichia*, *Clostridium*, *Fusobacterium* and *Actinobacillus* dominated in the microbiota of 1-day-old piglets. Although *Escherichia* is the first gut colonizer in different species [13, 14], *Fusobacterium* and *Actinobacillus* do not belong among commonly reported genera of the gut microbiome in warm blooded vertebrates. On the other hand, *Actinobacillus* and *Fusobacterium* colonize the palatine tonsils in pigs [15, 16] and their presence in the feces of young piglets might be therefore a consequence of their presence in the palatine tonsils, with limited multiplication along the poorly populated intestinal tract followed by fecal shedding. This is consistent with a report on *Actinobacillus’* low abundance in pig fecal microbiota [17]. The presence of *Fusobacterium* in fecal microbiota of very young piglets is of concern since *Fusobacterium* facilitates the development of swine dysentery [5,18,19].

In 4-day-old piglets, *Bacteroides* replaced *E*. *coli* as the most abundant microbiota member and *Fusobacterium* remained highly abundant, similar to a previous report [13]. The dominance of *Bacteroides* in the microbiota of 4-day-old piglets was rather unexpected since *Bacteroides* is not common in the fecal microbiota of adult pigs. Instead, *Prevotella* dominates over *Bacteroides* within phylum *Bacteroidetes* in adult pigs [8,13]. The cause for the transient appearance of *Bacteroides* is unclear. However, since the same observation has been already recorded [20], it seems to be a characteristic feature of microbiota development in piglets. An explanation can be sought in lactation, weaning and in changes in host gene expression. *Bacteroides* forage within the mucus covering gut epithelial cells [2] whose structure and composition change along the intestinal tract [21] and perhaps may change also with age. Moreover, IgA secretion into the gut lumen can be detected from day 21 of life with stable production from around day 30 of life [3] which may also affect microbiota composition.

The microbiota composition of adult sows at the phylum level (52% *Firmicutes*, 38% *Bacteroidetes* and 6% *Proteobacteria*) was similar to that reported previously [8, 22–24]. Within *Firmicutes*, the genera *Oscillospira* and *Ruminoccocus* were common both in sows and fattening pigs although *Lactobacillus* and *Clostridium* were present in the microbiota of fattening pigs at a much higher abundance [8] than in sows in this study. The extensive differences in microbiota composition in sows and piglets indicate that sows did not act as a major source of microbiota for piglets during the first days of life. Instead, breast feeding and environmental factors dominated over contact between sows and piglets as has been proposed previously [7]. However, we cannot exclude that skin, vaginal and breast milk microbiota may affect piglet microbiota what we did not address in this study. We also cannot exclude the role of sow as a donor of microbiota increases around the time of weaning which can be also influenced by different genetic background of each sow and piglet.

The differences in sow fecal microbiota kept under the two production conditions were moderate. Despite this, comparing the abundance of individual bacterial taxa showed that there were significant yet numerically not too extensive differences even among dominant genera. All 6 genera showing higher abundance in microbiota of sows kept under conventional conditions belonged to phylum *Firmicutes*. On the other hand, out of 7 genera exhibiting higher abundance in the microbiota of sows kept in enriched pens, 4 belonged to phylum *Bacteroidetes* and 1 to phylum *Fibrobacteres*. The representatives of *Bacteroidetes* and *Fibrobacteres* were repeatedly characterized as having the potential to metabolise non-soluble polysaccharides like cellulose, hemicellulose or pectin [25–27]. Since part of the enriched production system was a deep bedding of straw, the ingestion of straw enriched the feed with non-soluble polysaccharides and likely positively selected for the representatives of *Bacteroidetes* and *Fibrobacteres*. Although we did not address specifically microbiota composition in the cecum or colon, it is likely that microbiota in freshly collected fecal material correspond with the microbiota in distal parts of intestinal tract and may therefore influence sows metabolism and behavior.

In this study we have shown that the microbiota of sows kept under rearing conditions with or without a deep bedding of straw differed slightly with straw positively selecting for bacteria from phyla *Bacteroides* and *Fibrobacteres*. However, these differences were not reflected in the composition of microbiota of their piglets. The development of fecal microbiota in early days of piglet life was quite rapid and at least in the first days of life was not determined by sow microbiota. This is different from newly hatched chicken which can be easily populated by microbiota from adult hens [27]. It is likely the different feed composition delays the population of piglet intestinal tract by microbiota from adult sows. We therefore cannot exclude more important role of sows in shaping gut microbiota of their offspring around the time of weaning. In addition, microbiota colonizing skin or mammary gland were not analyzed in this study though it may also contribute the formation of gut microbiota in piglets.

## Supporting Information

S1 Table List of all bacterial genera identified in sow or piglet microbiota in this study. (XLS)
